# Comparison of Dorsal Quadratus Lumborum and Rectus Sheath Blocks for Canine Ovariectomy: A Randomised Clinical Trial

**DOI:** 10.3390/ani16213509

**Published:** 2026-11-06

**Authors:** Gonzalo Polo-Paredes, Sara Carrillo-Flores, Alberto Acosta, Xiomara Lucas, Francisco G. Laredo, Eliseo Belda

**Affiliations:** 1 Hospital Veterinario Universidad de Murcia, 30100 Murcia, Spain; gpolo@um.es (G.P.-P.); sacarriflores@gmail.com (S.C.-F.); alberto.a.u@um.es (A.A.); xiolucas@um.es (X.L.); laredo@um.es (F.G.L.); 2 Departamento de Medicina y Cirugía Animal, Facultad de Veterinaria, Universidad de Murcia, 30100 Murcia, Spain

**Keywords:** quadratus lumborum block, rectus sheath block, anaesthesia, analgesia, dogs, ovariectomy, neutering

## Abstract

This study aimed to compare the analgesic properties of the dorsal quadratus lumborum block (QLB) and the rectus sheath block (RSB) as part of a multimodal analgesic protocol in female dogs undergoing ovariectomy. A total of 57 dogs were included in the study. They were randomly divided into three groups: QLB, RSB or control. All the animals were premedicated with dexmedetomidine 0.125 mg m^−2^ and methadone 0.1 mg kg^−1^, induced with propofol and maintained with isoflurane. The blocks were performed using 0.4 mL kg^−1^ per hemiabdomen of bupivacaine 0.25%. In the intraoperative period, when the heart rate, mean arterial pressure or respiratory rate increased ≥ 20% from baseline, fentanyl 0.002 mg kg^−1^ was administered as rescue analgesia. During the early postoperative period, pain was assessed using the short form of the Glasgow Composite Pain Scale (GCPS-SF). Methadone 0.2 mg kg^−1^ was administered if GCPS-SF scores were ≥6/24 as rescue analgesia. No differences were found in fentanyl doses between groups (*p* = 0.4406). However, a clinically significant reduction of postoperative methadone rescue doses was found in the QLB group compared to the control group (26,32% absolute risk difference, with a 95% confidence interval of 6.64% to 49.10%), but not between QLB-RSB or RSB-control groups. In conclusion, dorsal QLB reduced early postoperative rescue analgesia requirements when included in a multimodal analgesic protocol in female dogs undergoing ovariectomy.

## Simple Summary

Ovariectomy (OVA) is a commonly performed surgical procedure in young female dogs. Although OVA is routinely performed, it remains an invasive procedure that requires appropriate analgesic management. Effective perioperative pain control improves patient recovery and reduces the risk of perianaesthetic complications. This study aimed to compare two regional anaesthetic fascial plane blocks, the quadratus lumborum block (QLB) and the rectus sheath block (RSB), as components of a multimodal analgesic protocol in dogs undergoing OVA. For this purpose, 57 female dogs scheduled for elective OVA were enrolled and randomly allocated to one of three groups: QLB, RSB or control. All groups received premedication with dexmedetomidine and methadone, anaesthesia was induced with propofol and maintained with isoflurane, and meloxicam was administered postoperatively. Bupivacaine 0.25% was used as the local anaesthetic for both the QLB and RSB techniques. Perioperative pain was assessed intraoperatively using physiological parameters and postoperatively with the Glasgow composite measure pain scale–short form for dogs. Fentanyl and methadone were administered as rescue analgesics during and after surgery, respectively, and analgesic requirements were compared among groups. No differences in intraoperative fentanyl requirements were observed between groups. Postoperatively, dogs in the QLB group required less analgesia than those in the control group; however, no differences were identified between the control and RSB groups or between the QLB and RSB groups. These findings suggest that the QLB improved postoperative analgesia compared with the control group.

## 1. Introduction

Ovariectomy (OVA) is a surgical technique designed to remove the female gonads to control small animal reproduction [1]. This surgery elicits a sympathetic, nociceptive and inflammatory response [2,3] that can be diminished by incorporating locoregional anaesthesia into multimodal analgesia protocols [4,5,6]. Locoregional anaesthesia techniques are an essential part of enhanced recovery after surgery (ERAS) protocols as they reduce hospitalization time, decrease anaesthesia and analgesia requirements [7] and lower perioperative morbidity/mortality [8].

The dorsal quadratus lumborum block (QLB) is a promising but under-evaluated technique firstly described by Alaman et al. [9] and Marchina-Gonçalves et al. [10] in canine cadavers. It aims to desensitise the rami ventrales and the rami communicantes of the last thoracic and first lumbar spinal nerves by injecting a local anaesthetic between the psoas muscle and the body of the first lumbar vertebra. This regional anaesthesia technique potentially provides analgesia to the middle and caudal abdominal wall and viscera.

Other injection points near these structures have been evaluated during female neutering. However, their analgesic efficacy has varied depending on the specific technique used. While clinical studies on lateral QLB approach during laparoscopic ovariectomies (lap-OVA) [4,5] and intermuscular QLB during ovariohysterectomies (OVH) [11] have confirmed their analgesic benefits, the outcomes of the intermuscular QLB during lap-OVA remain unclear [12].

The rectus sheath block (RSB) is a relatively recent technique reported in dogs [13,14] in which the local anaesthetic is administered between the epimisium of the rectus abdominis muscle and its internal sheath [13]. The objective of the RSB is to provide anaesthesia mainly to the ventrocranial abdominal wall by blocking the rami ventrales as they reach the rectus abdominis muscle near the linea alba.

To the authors’ knowledge, no clinical studies have compared these techniques in female dogs undergoing OVA. This clinical study aimed to compare the perianaesthetic analgesia provided by the dorsal QLB and the RSB as part of a multimodal analgesia protocol in dogs undergoing OVA. The need for rescue postoperative analgesia was defined as the primary outcome and the number of intraoperative rescue analgesic doses as the secondary outcome. Secondary objectives were to compare the incidence of perioperative hypotension, anaesthetic requirements and postoperative hypothermia.

We hypothesised that both dorsal QLB and RSB groups would show lower perioperative rescue analgesic requirements than a control group in which locoregional anaesthetic techniques were not employed.

## 2. Materials and Methods

### 2.1. Animals

This prospective, randomised and assessor-blinded study was approved by the Ethics Committee in Animal Experimentation of the University of Murcia (Registered number: CEEA-OH 967/2025).

Before the beginning of the study, we randomized the animals into three groups: quadratus lumborum block group (QLB group), rectus sheath block group (RSB group) and control group, in two distinct phases using randomisation software (www.randomizer.org). Throughout the two phases, dogs were assigned to their respective group in a strict order of inclusion into the study. Phase 1 consisted of a pilot study including 18 dogs (six per group) used to calculate sample size and were finally integrated into the final cohort of 57 animals (see Section 2.7*.* Sample size calculation). Subsequently, in Phase 2, the remaining 39 animals were randomly allocated into one of three groups (13 per group). The master randomization list was kept confidential, and the veterinarian responsible for the block checked the dog’s group assignment immediately after anaesthetic induction.

Female dogs admitted to the Hospital Veterinario Universidad de Murcia for elective OVA from March 2025 to March 2026 were selected for inclusion after a thorough anamnesis and clinical examination (thoracic auscultation, heart rate (HR), pulse quality, mucous membrane colour, capillary refill time, respiratory rate (RR) and pattern and rectal temperature). Animals eligible for inclusion were females aged between 6 months and 7 years, classified with a body condition score (BCS) between 3 and 7 out of 9 based on the WSAVA nutritional guidelines [15], with no evidence of clinical disease. Patients that were pregnant, brachycephalic, fractious, fearful or resistant to handling were excluded.

On the morning of the surgery, dogs were fasted for 8 h, and water was withheld 1 h before arrival. They were allowed to remain in a quiet room with their respective caregivers for acclimatisation until sedation.

### 2.2. Anaesthetic Protocol

A mixture of dexmedetomidine (0.125 mg m^−2^) (Dexdomitor 0.5 mg mL^−1^, Orion, Espoo, Finland) and methadone (0.1 mg kg^−1^) (Semfortan 10 mg mL^−1^, Dechra, Barcelona, Spain) was administered to all dogs in the muscular group formed by the semimembranous, and semitendinous muscles. Afterwards, animals were left undisturbed with their caregivers for 15 min.

Once sedation was achieved, the dogs were moved to the anaesthesia induction room. The same veterinarian (G.P.-P.) assessed and recorded the animals’ sedation depth using a sedation score ranging from 1 to 4 (Table 1).

Then, a catheter (16–22G) was placed in a cephalic vein, and an electrocardiogram (ECG) and non-invasive blood pressure (NIBP) (oscillometry) were obtained to verify the patient’s hemodynamic stability.

Before induction, the female dogs were preoxygenated for a minimum of 5 min using a tightly fitted mask connected to a Mapleson D circuit delivering 100% oxygen. Then, propofol (PropoVet Multidosis 10 mg mL^−1^, Zoetis, Madrid, Spain) was titrated to effect by 1 mg kg^−1^ bolus administered in 20 s. A period of 40 s was allowed between each propofol bolus to observe its effects. Intubation was attempted when the eyeballs were ventromedially rotated, and the palpebral reflex and jaw tone were absent. After intubation, dogs were connected to a rebreathing system, and isoflurane (Isoflo 100%, Zoetis, Madrid, Spain) administration was initiated and maintained between 1.2–1.5% expired fraction (EtIso). Dogs were allowed to breathe spontaneously.

Monitoring was performed using ECG, pulse-oximetry and plethysmography (SpO_2_), capnography and capnometry (EtCO_2_), oesophageal temperature, isoflurane inspired and expired fractions (Fi/EtIso) and oxygen inspired and expired fractions (Fi/EtO_2_) (General Electric B650 Monitor, GE HealthCare, Chicago, IL, USA), and NIBP (SunTech Vet25, SunTech Medical, Miami, FL, USA).

### 2.3. Locoregional Techniques

Afterwards, in the three groups, the lateral wall of the abdomen, caudad to the 12th rib, and the ventral abdominal wall of the dogs were clipped in the same way and thoroughly scrubbed with chlorhexidine (Clor-Scrub 4%, Imark-Hospital S.L., Madrid, Spain).

Then, in the QLB group, the animals were positioned in lateral recumbency and a dorsal approach to the QLB with 0.4 mL kg^−1^ of 0.25% bupivacaine (Bupivacaína, B Braun Medical S.A, Barcelona, Spain) was performed as previously described by Marchina-Gonçalves et al. [10]. The same procedure was performed in the contralateral hemiabdomen. The technique was considered successful when an anechoic pocket, corresponding to the local anaesthetic, spread around the quadratus lumborum and psoas minor or between these muscles and the vertebral body of L1.

In the RSB group, the dogs were positioned in dorsal recumbency and a RSB with 0.4 mL kg^−1^ of 0.25% bupivacaine was performed in each hemiabdomen according to the technique described by St. James et al. [13]. This technique was considered successful if an anechoic pocket spread between the internal rectus abdominis sheath, displaced dorsally, and the epimisium of that muscle, displaced ventrally. The total dose of bupivacaine was 2 mg kg^−1^ in both groups.

Both techniques were performed with a 3–13 MHz linear ultrasound probe (SL1543, MyLabGamma Vet, Esaote, Florence, Italy) and a sonovisible needle (Ultraplex 20 G, 100 mm, 30°, BBraun, Melsungen, Germany.

Finally, in the control group, the animals were clipped, but no locoregional anaesthetic techniques were performed.

Following thorough surgical scrubbing, the patients were transferred to the surgical room and monitored as previously described. All locoregional block techniques were performed by the same veterinary anaesthetist (G.P.-P.).

### 2.4. Surgical Technique

A 20-min period was strictly maintained between the end of the locoregional block and the surgical incision. In the control group, a 20-min period, starting at the end of hair clipping, was also maintained. Thereafter, a midline laparotomy was performed with a scalpel, the ovaries were located and excised using an *en bloc* technique with a bipolar electrosurgical device (LigaSure^®^ and LigaSure Impact^®^, Medtronic, Auckland, New Zealand). Then, the abdominal wall was closed in layers (muscular and intradermic layers) using a continuous suture pattern with an absorbable monofilament suture (Monosyn, B-Braun VetCare S.E, Barcelona, Spain). Surgery duration was measured and defined as the time from skin incision to completion of the intradermal suture. All surgical procedures were performed by the same surgeon (A.A.).

### 2.5. Intraoperative Period

Immediately after endotracheal intubation, the aforementioned parameters (ECG, SpO_2_, EtCO_2_, temperature, NIBP, Fi/EtIso and Fi/EtO_2_) were recorded every 10 min. Jaw tone, eyeball position and absence of palpebral reflex were also evaluated to assess anaesthetic depth at 10-min intervals.

Once in the surgical room and following the placement of surgical drapes, a second veterinary anaesthetist (S.C.-F.), unaware of the analgesia strategy employed, assumed supervision of the anaesthesia and clinical decision-making.

During the procedure, five surgical time points were registered: (T1) incision on the skin, (T2) traction of the first ovary, (T3) traction of the second ovary, (T4) suture of the muscular and (T5) intradermal layer.

Before incision, a baseline HR, RR and MAP were recorded. Nociception was defined as an increase of 20% or greater in at least one of these parameters within one minute, while confirming a surgical depth of anaesthesia (absence of jaw tone and palpebral reflex, and ventromedial eye rotation). In the presence of a nociceptive phenomenon, an IV bolus of fentanyl (0.002 mg kg^−1^) (Fentadon 50 microgramos mL^−1^, Dechra, Barcelona, Spain) was administered. The number of fentanyl doses required during surgery and the surgical time point at which they occurred were registered.

Hypotension, defined as MAP of <60 mmHg, was treated at the discretion of the veterinarian with either ephedrine 0.1 mg kg^−1^ IV or atropine 0.02 mg kg ^−1^ IV. The drug, number of doses and timing of administration were recorded. Once the hypotension was corrected, the procedure resumed.

### 2.6. Postanaesthetic Period

Endotracheal extubation was attempted once a swallowing reflex was observed, and the time of extubation was registered. At that point, a thorough clinical exam was again performed to assess immediate postanaesthetic complications. Anaesthesia duration was defined as the time from anaesthetic induction to tracheal extubation, and the presence of postanaesthetic hypothermia was defined as a rectal temperature below 37.5 °C. Immediately after recovery, meloxicam (0.2 mg kg^−1^) IV (Canidolor 5 mg mL^−1^, Fatro, Barcelona, Spain) was administered.

Animals were then transferred to the hospitalisation area for postoperative monitoring. Dogs were placed in a clean, dry and comfortable environment, and hypothermia, if present, was corrected using infrared heat lamps.

Postanaesthetic pain was assessed using the canine short form of the Glasgow Composite Pain Scale (GCPS-SF) [16] at 1, 2, 4 and 6 h after endotracheal extubation. Pain was defined as a score of ≥6/24. In that case, a bolus of methadone (0.2 mg kg^−1^) IV was administered, and the postanaesthetic timing and number of doses were recorded.

All the pain scale evaluations were performed by the same anaesthetist who made the intraoperative evaluations and was blind to the analgesic protocol of the animals (S.C.-F).

Any other complication during this period was also assessed. Seven hours after endotracheal extubation, animals were discharged from the hospital unless pain, hypotension or hypothermia were still observed.

### 2.7. Sample Size Calculation

The primary outcome was defined as the number of animals that achieve freedom from postoperative rescue analgesia among the three experimental groups. The sample size was calculated with an online software (GRANMO, Institut Hospital del Mar d’Investigacions Mèdiques, Barcelona, Spain). To ensure adequate statistical power across the trial, the sample size estimation was based on a two-sided pairwise comparison between the two extreme groups (control and QLB). Assuming an alpha risk of 0.05, a statistical power of 0.8 and a 0% drop-out rate, a minimum of 19 animals per group (a total of 57 for the three groups) was required to detect significant differences.

This calculation was based on a preliminary pilot study involving 18 animals (six per group). GRANMO used the standard normal deviate test for the difference between two independent proportions using the arcsine transformation formula:

$$n\;=\;\frac{2\;\cdot \;{\left(Z_{\propto /2}\;+\;Z_{\beta }\right)}^{2}}{{\left[2\;\cdot \;\arcsin\left(\sqrt{p_{2}}\right)\;-\;2\;\cdot \;\arcsin\left(\sqrt{p_{1}}\right)\right]}^{2}}$$

where n represents the sample size per study group, Z_∝/2_ is the critical value for two-tailed significance level (1.96), Z_β_ is the critical value for the Type II error (0.84), and p_1_ and p_2_ are the expected proportions in the control and experimental groups, respectively. p_1_ = 0.80 and p_2_ = 1.00. The assumed absolute effect size was 20%, and the variability/events rate was calculated as 0.16 for the control group and 0 for the QLB group.

The data from the 18 pilot animals were fully included within the final study population of 57 dogs.

### 2.8. Statistical Analysis

Statistical analysis was performed using R version 4.4.2 (R: A language and environment for statistical computing, R foundation for statistical computing, Vienna, Austria, https://www.R-project.org/).

Data were first tested for normality using the Shapiro–Wilk test, and then subsequently assessed independently for each group. For descriptive statistics, data were expressed as mean ± SD for parametric variables, and median [Interquartile Range (IQR)] for non-parametric variables.

The weight and rectal temperature at extubation were analysed using a one-way ANOVA.

A Kruskal–Wallis test and posterior Dunn Test with Bonferroni correction, if needed, were performed for age, BCS, sedation score, surgical and anaesthetic duration, dose of propofol, number of doses of fentanyl and methadone and GCPS-SF score values at 1, 2, 4 and 6 h.

Categorical variables (presence or absence of intraoperative hypotension and postanaesthetic hypothermia and time of nociceptive event) were expressed as percentages and frequencies. We first analysed the expected frequencies for each variable; a chi-square test was performed when all expected frequencies were ≥5, otherwise, Fisher’s exact test was used. Due to the binary nature of the postoperative methadone rescue doses (0–1) data were analysed as a categorical variable (yes/no) using the Fisher–Freeman–Halton test. Subsequently, pairwise comparisons were performed using the Boschloo’s unconditional exact test with Holm–Bonferroni correction due to the small sample size and zero-cell counts. Finally, clinical effect sizes were quantified using absolute risk differences (ARD) with their corresponding 95% confidence intervals (CI) calculated using the Wilson–Newcombe score method. Additionally, to objectively evaluate a possible clinical spectrum across groups, a Cochran–Armitage trend test was conducted with the groups ordered from the lowest to highest theoretical analgesic efficacy (control–RSB–QLB).

Finally, the EtIso concentrations were compared between groups using a linear mixed model (LMM). In this model, EtIso was defined as the continuous dependent variable, while treatment group, time of anaesthesia and their interaction were introduced as fixed effects. To account for within-subject correlation and individual variability, a random intercept was specified for each patient.

For all statistical tests, the significance level was set at *p* < 0.05.

## 3. Results

A total of 81 animals were assessed for eligibility, of which 22 were excluded. Five animals failed to attend their surgical appointment, 11 owners refused to participate in the study, and six animals did not meet the inclusion criteria due to brachycephalic breed (n = 2), obesity with a BSC of 8/9 (n = 1) and systemic disease (n = 3; two with advanced degenerative mitral valve disease and one with uncontrolled hypothyroidism).

Finally, 59 animals met the inclusion criteria and underwent randomisation. During the pilot phase (Phase 1), blood was detected during the pre-injection aspiration phase in two animals allocated to the QLB group. Bupivacaine administration was cancelled, and the animals were excluded from the study. To maintain group balance, these two excluded animals were immediately replaced by the next consecutive eligible patients admitted to the hospital, who were assigned to the same group following the sequential protocol until a final sample size of 57 animals successfully completed the study (Table 2). To maintain the assessor-blinded design of the study, the blinded evaluator was not informed of the reasons for animals’ exclusions until the completion of the study. The excluded animals underwent OVA without encountering anaesthetic or surgical complications.

The blocks in the remaining animals were considered successful. Consequently, the overall block success rate was 90.47% (19/21 dogs) in the QLB group and 100% (19/19 dogs) in the RSB group.

No additional animals were dropped from the study, and no losses to follow-up were encountered (Figure 1).

The animals were 24 [7–84] months of age, weighed 15.6 [3.08–37.60] kg, and had a BCS of 4 [3–7]. No statistical differences were found between groups regarding age and weight. A statistically significant difference was observed between control and RSB groups regarding BCS.

No statistical differences were found in surgery and anaesthesia times, sedation scores, total propofol dose, rescue fentanyl doses, GCPS-SF at 1, 2, 4 and 6 h, temperature at endotracheal extubation, presence or absence of hypothermia or hypotension or EtIso between groups. The drugs used to treat hypotension and the group assignment of the treated animals are shown in Table 3.

Intraoperative rescue analgesia was required in 52.63% (10/19), 47.4% (9/19) and 31.6% (6/19) of dogs in the control, RSB, and QLB groups, respectively. The dogs had a nociceptive stimulus at T2 or T3 (first or second ovary retraction), with no differences between groups in the timing of the stimulus (*p* = 0.4393). No dogs received two concurrent doses at any single time point; however, consecutive fentanyl boluses were administered at T2 and T3 to one dog in the control group, one dog in the QLB group and two dogs in the RSB group. The relationship between the surgery time point and the administration of fentanyl doses can be found in Table 4.

Regarding postoperative analgesia, none of the patients (0%—0/19) in the QLB group needed postoperative methadone. On the other hand, 10.53% (2/19) of RSB and 26.32% (5/19) of control group dogs needed rescue analgesia. The Fisher–Freeman–Halton test did not reveal statistical significance (*p* = 0.057). However, the pairwise post-hoc analysis revealed a clinically significant difference between the QLB and the control groups, with an ARD of 26.32% (95% CI 6.94–49.10%; unadjusted *p* = 0.023). Although the subsequent Holm–Bonferroni correction resulted in a marginal tendency without reaching conventional statistical significance (adjusted *p* = 0.070), the confidence interval strictly excludes zero. No clinical or statistical differences were found between the remaining pairwise comparisons (QLB vs. RSB and RSB vs. control groups). Additionally, the Cochran–Armitage test demonstrated an ordered clinical spectrum among groups (Chi-square for trend = 6.107, *p* = 0.0135).

Seven hours after orotracheal extubation, all 57 animals were discharged with no evidence of hypothermia or hypotension. Statistical results are summarised in Table 5. The proportion of dogs receiving postoperative analgesia and their confidence interval are represented in Figure 2.

## 4. Discussion

This study showed that the dorsal QLB reduces postoperative analgesic requirements in female dogs submitted to OVA when included in a multimodal analgesia plan. Although conventional statistical significance was not reached (*p* = 0.070), probably underpowered by the small sample size, the post-hoc pairwise comparison demonstrated a clinical benefit, evidenced by an ARD of 26,32% and a 95% CI of 6.94 to 49.09%. The fact that the 95% CI strictly excludes zero supports a meaningful clinical reduction in postoperative methadone requirements in the QLB group.

These results are consistent with those previously reported for lateral QLB in female dogs submitted to laparoscopic OVA [4,5] or for intermuscular QLB and OVH [11,17]. However, the present study failed to detect any differences between locoregional techniques.

Our findings suggest that there is a gradual clinical spectrum across QLB, RSB and control groups based on the Cochran–Armitage trend test results (*p* = 0.0135) and the percentage of animals requiring early postoperative rescue analgesia (0%, 10.53 and 26.32%, respectively). Cadaveric studies performed in dogs showed that dorsal QLB reaches both visceral and somatic pain transmission pathways [9,10], whereas RSB reaches solely somatic fibres [13]. This difference could explain the gradual clinical responses observed in the postoperative period.

In contrast, our results showed no differences in intraoperative rescue analgesia between groups. Female dogs that required fentanyl exhibited a nociceptive response during the traction of the first or the second ovary. These organs receive sensory innervation from the tenth thoracic (T10) to the fourth lumbar segment (L4) [18]. The aforementioned QLB cadaveric studies showed consistent staining of the sympathetic trunk between the thirteenth thoracic (T13) and the third lumbar segment (L3), and an erratic staining craniocaudally with an anecdotal staining of T10. These findings could explain the inconsistent analgesia of dorsal QLB during ovarian traction, as the local anaesthetic may not block all the nerves mediating ovarian pain perception. Furthermore, clinical studies in humans have suggested that the area of desensitisation achieved with locoregional techniques expands over time [19]. This phenomenon could also occur in dogs, potentially explaining the enhanced analgesia provided by dorsal QLB in the postoperative period.

Previous studies have assessed differences in the efficacy of locoregional techniques used either during OVA or OVH. Contrary to our results, Fumanelli et al. [12] found no difference in perioperative rescue analgesia between the control group, the intermuscular QLB and the transversus abdominis plane (TAP) block. The latter block aims to desensitise the rami ventrales of the last thoracic and first lumbar spinal nerves by injecting the local anaesthetic between the transversus abdominis and the internal oblique muscles. TAP block seems to provide unreliable analgesia to the ventral midline in human volunteers, while producing reliable desensitisation of the lateral abdominal wall [20]. Therefore, RSB might be a better option. Cadaveric studies performing RSB in dogs showed consistent staining of the rami ventrales between T10-T13 and erratic staining of L1, potentially providing analgesia to the cranial abdominal wall region but rarely to the middle abdominal region [13], which is the area of the surgical incision. This fact could explain the lack of differences between the RSB and control groups. In contrast, cadaveric studies performing TAP block showed consistent staining of the medial and caudad region of the abdomen (T13-L3) [21,22], potentially providing analgesia to the region where the incision is performed in OVAs. Despite the aforementioned evidence, direct comparison between TAP block and RSB showed no difference in perioperative rescue analgesia or postoperative GCPS-SF in dogs undergoing OVH [14]. Hence, the clinical advantages of any technique over the others remain unknown.

In our study, the lack of intraoperative differences in the fentanyl requirements between groups may be attributed to the high efficacy of the multimodal analgesic protocol. Our multimodal strategy included several analgesic drugs. Dexmedetomidine, an alpha-2 receptor agonist, exerts its analgesic action at the level of the spinal dorsal horn and at the locus coeruleus of the brainstem [23]. Its analgesic and sedative effects have been shown to follow an allometric scale [24]. Based on this property, and due to the variety of size of dogs in our study, dexmedetomidine was dosed according to body surface area rather than body weight. Additionally, a recent study performed in female dogs submitted for laparoscopic ovariectomies showed excellent analgesia when dexmedetomidine and its constant rate infusion were included in an opioid free analgesia protocol [25]. Methadone is a full mu opioid receptor agonist that has also agonistic monoamine and antagonistic NMDA effects both in peripheral and central tissues [26]. Methadone has shown better analgesic properties than the partial mu opioid receptor buprenorphine in ovariohysterectomies in dogs [27]. Moreover, the combination of dexmedetomidine and low doses of the full mu opioid morphine showed superior postoperative analgesia to high doses of morphine alone [28]. Similar behaviour might be expected from methadone, considering its comparable pharmacodynamic characteristics. Under general anaesthesia and with these conditions, the additional contribution of a locoregional technique might not suffice to demonstrate statistical significance, despite providing a clinical improvement, mainly in the postoperative period.

Meloxicam, a preferential COX-2 inhibitor, could also play an important role in postoperative analgesia. When given preoperatively in dogs submitted to combined laparoscopic OVA and laparoscopic gastropexy, meloxicam has shown a reduction in postoperative pain scores compared to robenacoxib in a dexmedetomidine–methadone–sufentanil analgesic protocol [29]. Furthermore, during OVH, meloxicam has shown better analgesic properties than butorphanol [30] or carprofen [31].

Regarding the complications encountered, blood was observed during aspiration when performing the dorsal QLB in two dogs. This complication may be due to inadvertent puncture of the lumbar arteries, a branch of the aorta that runs next to the vertebral bodies. Even though these animals were excluded from the study, the OVA was still performed. No intraoperative or postoperative complications were encountered in these animals. Retroperitoneal haematoma has been previously reported in dogs [32] and cats [33] after dorsal QLB. While needle aspiration was negative in these case reports, we observed blood in the couple of animals excluded, pointing out the relevance of aspiration before injecting to rule out the IV administration of local anaesthetics. Indeed, inadvertent vascular injection or exceeding the recommended doses of local anaesthetics can result in cardiac and neurologic toxicity [34]. To mitigate this risk, the total dose used in this study was 2 mg kg^−1^ of bupivacaine for both blocks, which is considered the maximum recommended dose for this drug [35]. In addition, a lower concentration of bupivacaine (0.25%) was used to increase the total volume without exceeding the recommended dose. However, it should be noted that a reduction of the concentration of a local anaesthetic could result in a reduction of the time of action [36].

Isoflurane requirements did not significantly differ between groups. Under these circumstances, and despite the dorsal QLB can potentially block a large extension of the sympathetic trunk, the QLB group did not show a higher incidence of hypotension compared to the RSB and the control groups. In our study, all hypotensive episodes took place before the incision. Hypotension was immediately corrected. However, the administration these drugs required a readjustment of the baseline values used to assess intraoperative nociception. Once hypotension was corrected, HR and MAP values were assessed every two minutes. When two consecutive measures showed no significant changes (less than 10%), stabilisation of the corrective drug effects was assumed, and new HR, RR and MAP baselines were recorded at that moment and surgery commenced thereafter. Despite this corrective treatment, sympathetic responses to surgical nociception remained clearly identifiable, allowing us to include these patients in the analysis.

Our study has several limitations. Firstly, the intraoperative evaluation was limited to a threshold-based assessment of nociception (>20% deviation from baseline). Because the peak-to-baseline ratio was not assessed, we could not determine whether either technique was superior at blunting the severity of the autonomic response. Secondly, only bupivacaine was evaluated in our study; differences in pharmacodynamics, pharmacokinetics, and nerve fibre preferential blocking between local anaesthetics drugs may vary the results [36]. Thirdly, the study was not completely blind; even though all animals were clipped the same way, the locoregional techniques could have left a visible mark at the puncture site. In addition, we decided not to include a locoregional control group (QLB or RSB injecting saline) due to ethical concerns; the animals were first and foremost patients from our hospital and performing a puncture without potential clinical benefit might present ethical issues. Fourthly, atropine or ephedrine were administered to treat hypotension. Their anticholinergic and sympathomimetic actions, respectively, could have masked nociceptive increases in HR, RR or MAP. Fifthly, only the GCPS-SF was used to compare postoperative analgesia. Determining the mechanical nociceptive threshold or analysing stress and inflammation parameters may have revealed subtle differences between techniques. In addition, the researcher who performed intraoperative nociceptive assessment also performed the postoperative GCPS-SF, intraoperative nociception levels could have influenced the evaluator to over- or under-diagnose postoperative pain, despite the use of validated pain scales, because outcomes depend on subjective interpretation of the patient’s behaviour. Sixthly, postoperative sedation depth was not scored; thus, high sedation levels may have confounded the first GCPS-SF assessments. Finally, the postoperative evaluation was limited to the first 6 h, although all animals exhibited very low GCPS-SF values at that time point, subsequent differences among groups thereafter may have been overlooked.

## 5. Conclusions

In our study, the addition of a dorsal QLB to a multimodal analgesia strategy demonstrated a clinically meaningful reduction in early postoperative rescue analgesia requirements in dogs undergoing elective ovariectomy when compared with systemic analgesia. No statistically significant reduction in rescue analgesia was detected for RSB compared with control. In addition, no significant differences in intraoperative rescue analgesia, hypotension or postoperative hypothermia were found between groups. Further studies with larger sample sizes are warranted to definitively establish the statistical superiority of QLB.

## Figures and Tables

**Figure 1:**
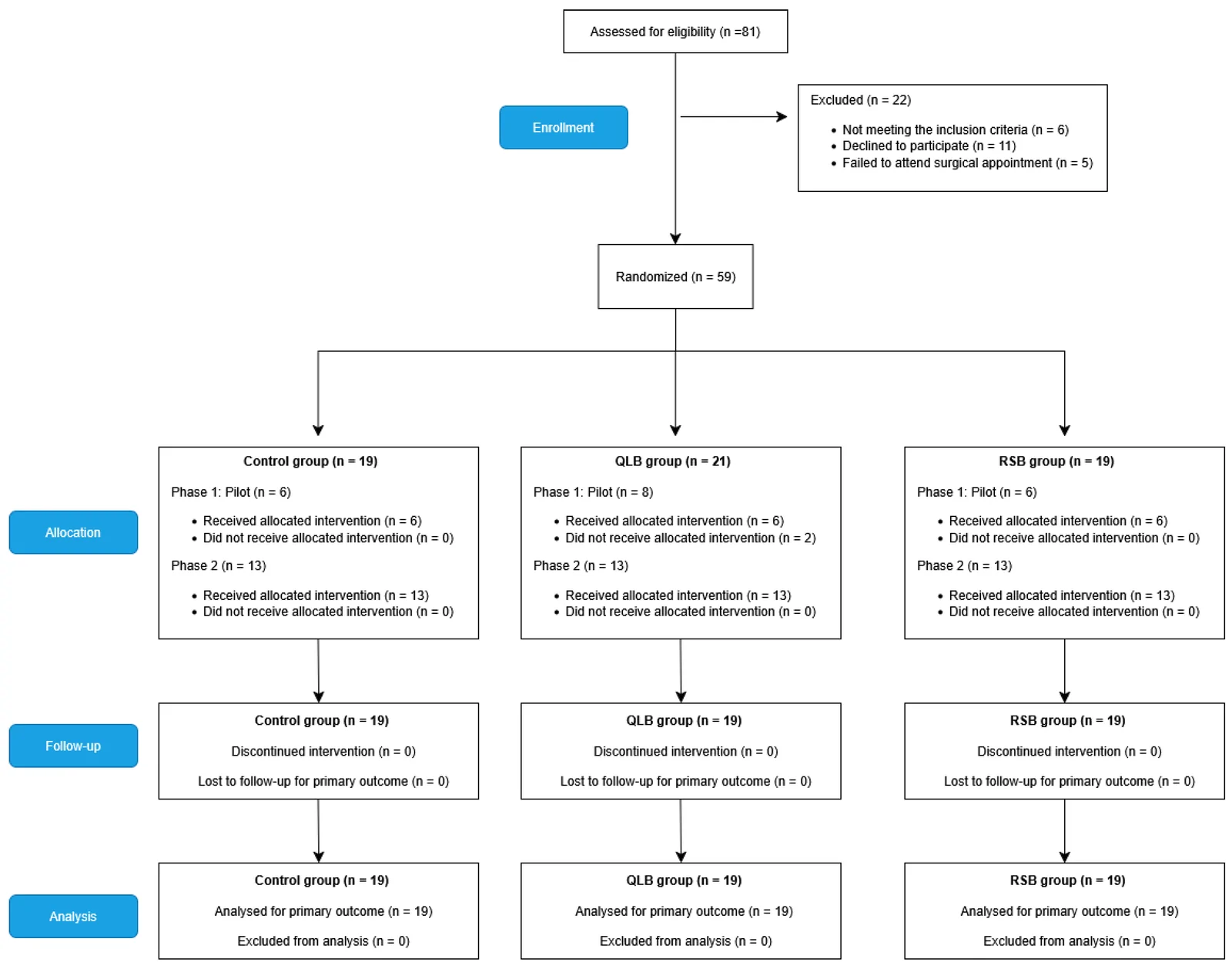
CONSORT flow diagram showing the distribution of animals during the study. The trial was divided into two phases: Phase 1, consisting of a pilot study whose data were used to calculate sample size, and Phase 2, the definitive study. Data from animals in Phase 1 were pooled with Phase 2 for the final outcome analysis.

**Figure 2:**
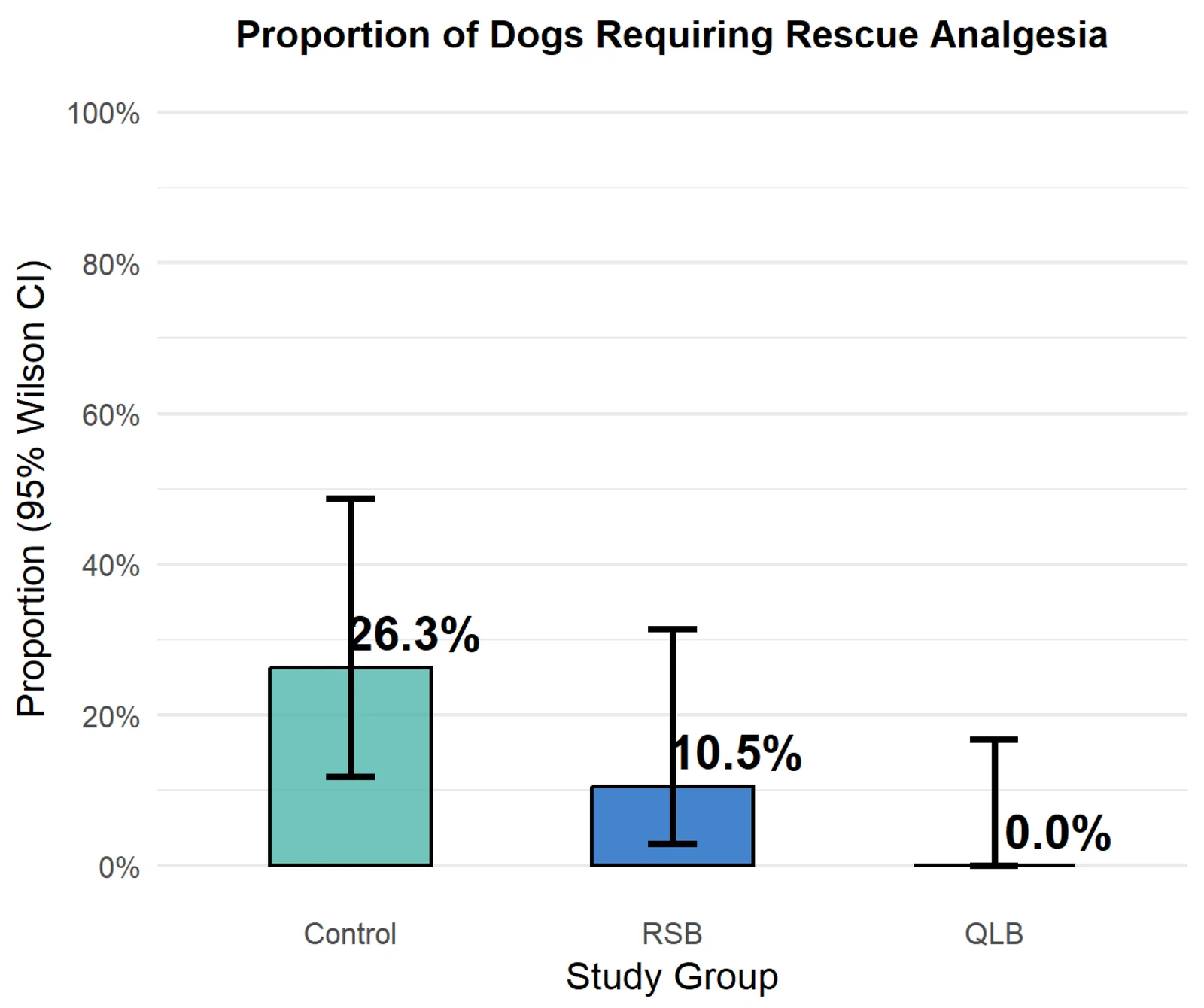
Proportion of dogs requiring postoperative rescue analgesia in dogs submitted to ovariectomy. Bars represent the proportion of patients requiring methadone rescue in the control, rectus sheath block (RSB), and quadratus lumborum block (QLB) groups. Error bars denote the 95% confidence intervals (CI) calculated using the Wilson score method. Lower and upper CIs: control group (11.81–48.79%), RSB (2.94–31.39%) and QLB (0.00–16.82%). Data labels indicate the exact percentage within each group.

**Table 1:** Simple descriptive scale for the qualitative assessment of sedation depth in dogs based on posture and response to stimuli.

Sedation Score	Criteria
1	Not sedated: ambulating or standing, responsive to auditory or visual stimuli.
2	Ataxic but able to stand, responsive to auditory or visual stimuli.
3	Sternal or lateral recumbency, responsive to auditory or visual stimuli.
4	Sternal or lateral recumbency, unresponsive or highly reduced responses to auditory or visual stimuli.

**Table 2:** Breed distribution of the dogs included in the study.

Breed	Number of Animals
Mixed breed	28
Spanish Greyhound (Galgo)	5
Andalusian Terrier (Bodeguero Andaluz)	4
American Stafford	3
Pinscher	3
Spanish Water Dog	2
Chihuahua	2
Brittany Spaniel	2
German Shepherd	2
Weimaraner	1
Border Collie	1
Australian Shepherd	1
Murcian Ratter (Ratonero murciano)	1
Labrador	1
Maltese	1
Total	57

**Table 3:** Drugs used to treat hypotension and the group assignment of the treated animals.

Group	Drug Used to Treat Hypotension	Number of Animals Treated
Control	Atropine	3
Control	Ephedrine	3
QLB	Atropine	1
QLB	Ephedrine	1
RSB	Atropine	2
RSB	Ephedrine	3

**Table 4:** Distribution of fentanyl rescue doses administered per study group during specific intraoperative time points.

Surgical Time Point	Clinical Intervention	Control Group (Doses)	QLB Group (Doses)	RSB Group (Doses)
T1	Skin incision	0	0	0
T2	Traction of 1st ovary	7	4	7
T3	Traction of 2nd ovary	4	3	4
T4	Muscular layer suture	0	0	0
T5	Intradermal suture	0	0	0
		Total: 11	Total: 7	Total: 11

**Table 5:** Descriptive statistics and differences in physiologic and anaesthesia-related parameters between female dogs submitted to elective ovariectomy receiving locoregional anaesthesia within a multimodal analgesia protocol. Statistical significance was set at *p* < 0.05. QLB, Quadratus lumborum group; RSB, Rectus sheath group.

Parameter	Control	QLB	RSB	*p*-Value	Post Hoc Control vs. QLB	Post Hoc Control vs. RSB	Post Hoc QLB vs. RSB
Weight (kg)	15.1 ± 7.55	15.3 ± 9.10	16.4 ± 10.6	0.896	-	-	-
Age (months)	30 [16.5–43]	18 [12–31.5]	18 [12–34]	0.2643	-	-	-
BCS (/9) *	4	4	5 [4–5]	0.0391 *	0.9909	0.0418 *	0.0813
Sedation score	4 [3–4]	3 [3–3.5]	3 [3–4]	0.0815	-	-	-
Propofol dose (mg/kg)	2 [1–2]	2 [1–2]	2 [1–2]	0.882	-	-	-
Fentanyl (number of doses)	1 [0–1]	0 [0–1]	0 [0–1]	0.4406	-	-	-
Surgery time (min.)	16 [14–20]	14 [10–17]	15 [11.5–20]	0.1606	-	-	-
Anaesthesia time (min.)	61 [ 58–68.5]	61.5 [55.8–73]	70 [63.5–74.5]	0.0536	-	-	-
Methadone doses (Number of animals requiring methadone [%])	0 [0–0.5] (26.32%)	0 [0] (0%)	0 [0] (10.53%)	0.0574	0.0704 (ARD: 26.32%, CI: 6.94–49.09)	0.5117 (ARD: 15.78%, CI: −10.15–40.94)	0.5117 (ARD: −10.52%, CI: −31.73–7.61)
GCPS-SF 1 h (/24)	3 [2.5–5]	3 [3]	3 [2–4]	0.3388	-	-	-
GCPS-SF 2 h (/24)	2 [1–3.5]	2 [1–2.5]	2 [1–3]	0.5637	-	-	-
GCPS-SF 4 h (/24)	2 [1–2]	2 [1–2.5]	1 [1–2]	0.3385	-	-	-
GCPS-SF 6 h (/24)	1 [1–1.5]	1 [0.5–2]	1 [0.5–2]	0.8936	-	-	-
Temperature at extubation (°C)	37.5 ± 0.77	37.1 ± 0.96	37.2 ± 0.781	0.287	-	-	-
Hypothermia (yes/no)	42.1% (8/19)	63.15% (12/19)	47.36% (9/19)	0.3333	-	-	-
Hypotension (yes/no)	31.57% (6/19)	10.52% (2/19)	26.31% (5/19)	0.3757	-	-	-
EtIso (%)	1.2 [1.0–1.3]	1.1 [1.1–1.3]	1.2 [1.1–1.4]	0.3929	-	-	-

* Statistical significance (*p* < 0.05).

## Data Availability

Data supporting the reported results can be sent to anyone interested by contacting the corresponding author.

## Abbreviations

The following abbreviations are used in this manuscript:

OVA	Ovariectomy
ERAS	Enhanced recovery after surgery
lap-OVA	Laparoscopic ovariectomy
QLB	Quadratus lumborum block
OVH	Ovariohysterectomy
RSB	Rectus sheath block
BCS	Body condition score
HR	Heart rate
RR	Respiratory rate
ECG	Electrocardiogram
NIBP	Non-invasive blood pressure
SpO_2_	Peripheral oxygen haemoglobin saturation
EtCO_2_	End-tidal carbon dioxide pressure
Fi/EtO2	Inspired and expired oxygen fraction
EtIso	Expired isoflurane fraction
ARD	Absolute risk difference
CI	Confidence interval
